# Effect of *Matricaria aurea* Essential Oils on Biofilm Development, Virulence Factors and Quorum Sensing-Dependent Genes of *Pseudomonas aeruginosa*

**DOI:** 10.3390/ph17030386

**Published:** 2024-03-18

**Authors:** Haitham Qaralleh, Sultan Ayesh Mohammed Saghir, Muhamad O. Al-limoun, Saif M. Dmor, Khaled Khleifat, Basma Ezzat Mustafa Al-Ahmad, Laila Al-Omari, Yasser Tabana, Ramzi A. Mothana, Hanan M. Al-Yousef, Abdulaziz M. Alqahtani

**Affiliations:** 1 Department of Medical Laboratory Sciences, Mutah University, Mutah 61710, Jordan; alkhkha@mutah.edu.jo; 2 Department of Medical Analysis, Princess Aisha Bint Al-Hussein College of Nursing and Medical Sciences, Al-Hussein Bin Talal University, Ma’an 71111, Jordan; sultan.s.ayesh@ahu.edu.jo (S.A.M.S.); seif.aldmour@ahu.edu.jo (S.M.D.); 3 Department of Biological Sciences, Faculty of Science, Mutah University, Mutah 61710, Jordan; moallimoun@mutah.edu.jo; 4 Department of Fundamental Dental Medical Sciences, Kulliyyah of Dentistry, International Islamic University Malaysia, Kuantan 25200, Pahang, Malaysia; drbasma@iium.edu.my; 5 Faculty of Allied Medical Sciences, Al-Ahliyya Amman University, Amman 19111, Jordan; l.omari@ammanu.edu.jo; 6 Faculty of Pharmacy and Pharmaceutical Sciences, University of Alberta, Edmonton, AB T6G 2E1, Canada; tabana@ualberta.ca; 7 Department of Biochemistry, University of Alberta, Edmonton, AB T6G 2R3, Canada; 8 Department of Pharmacognosy, College of Pharmacy, King Saud University, Riyadh 11451, Saudi Arabia; rmothana@ksu.edu.sa (R.A.M.); halyousef@ksu.edu.sa (H.M.A.-Y.); abmalqahtani@ksu.edu.sa (A.M.A.)

**Keywords:** quorum sensing, biofilm, *Matricaria aurea*, α-bisabolol oxide A, *Pseudomonas aeruginosa*, virulence factors, *phazA1*, *aprA*

## Abstract

The emergence of drug-resistant microorganisms presents a substantial global public health threat. The increase in pathogens resistant to commonly prescribed antibiotics underscores the urgent requirement to explore alternative treatment strategies. This study adopts a novel approach by harnessing natural resources, specifically essential oils (EO), to combat bacterial pathogenicity. The primary aim of this research was to analyze the chemical composition of the aerial part of the *Matricaria aurea* (*M. aureas*) EO and evaluate its potential for inhibiting quorum sensing (QS) and disrupting biofilm formation in *Pseudomonas aeruginosa* (*P*. *aeruginosa*). The gas chromatography-mass spectrometry (GCMS) analysis unveiled that α-bisabolol oxide A constituted the predominant portion, comprising 64.8% of the total, with β-bisabolene at 6.3% and α-farnesene at 4.8% following closely behind. The antibiofilm efficacy was observed at concentrations of 0.3, 0.15, and 0.08 mg/mL, demonstrating negligible effects on cell viability. Furthermore, the EO from *M. aurea* effectively inhibited the formation of *P. aeruginosa* biofilms by diminishing aggregation, hydrophobicity, and swarming motility. Significantly, the EO treatment resulted in a conspicuous decrease in the production of pyocyanin, rhamnolipid, and extracellular polymeric substances (EPS), along with a reduction in the enzymatic activity of protease and chitinase. The EO effectively hindered QS by disrupting QS mechanisms, resulting in a marked decline in the secretion of N-Acyl homoserine lactone (AHL) molecules and the expression of *phazA1* and *aprA* genes. This investigation offers compelling evidence supporting the potential of *M. aurea* EO as a promising therapeutic candidate for addressing infectious diseases induced by biofilm formation.

## 1. Introduction

*Pseudomonas aeruginosa* (*P*. *aeruginosa*) stands as the principal causative agent behind nosocomial pneumonia and respiratory failure [1]. Individuals with compromised immune systems, such as those living with Acquired Immunodeficiency Syndrome (AIDS), are experiencing a notable increase in life-threatening infections linked to *P*. *aeruginosa* [1]. This bacterium plays a pivotal role in promoting bacteremia and sepsis among patients undergoing chemotherapy [2]. Infections caused by *P. aeruginosa* often affect individuals with diabetic ulcers, burn injuries, corneal ulcers, and surgical wounds [3]. Additionally, individuals with cystic fibrosis (CF) often face persistent *P. aeruginosa* infections, which play a pivotal role in the progression of pulmonary failure and, tragically, can result in fatalities for these patients [4]. Moreover, the ability of *P. aeruginosa* to produce extracellular enzymes frequently categorizes it as a prevalent spoilage microorganism, especially in food products recognized for their high water content and rich nutrients [3,4].

In the 1960s, when *P. aeruginosa* initially emerged as a significant cause of septicemia, the mortality rate was approximately 90% [5]. The advent of antibiotics marked a significant advancement in the treatment of *P. aeruginosa* bacteremia, yet it continues to pose a significant infection risk, associated with considerable morbidity ranging from 18% to 61% and a persistent mortality rate [6,7,8]. Adding to the challenge of combating infections caused by this bacterium is its robust inherent resistance as well as acquired resistance to a wide array of currently available antibiotics [9,10]. Given the formidable nature of this challenge and the severity of *P. aeruginosa* infections, it has been designated as one of the priority pathogens within the group known as ESKAPE, underscoring the urgent demand for potent antibacterial agents to address this threat [9,11]. Researchers are continually investigating novel agents that can overcome antibiotic resistance mechanisms while minimizing harm to the host. The quest for novel anti-quorum sensing (QS) agents is of considerable interest, given the diverse avenues available [12]. Proficient QS inhibitors diminish the pathogenicity of *P. aeruginosa* and curtail its capacity to induce disease, thereby enabling the immune system to efficiently combat the infection [13].

*P. aeruginosa* employs a QS system, a regulatory mechanism, to synchronize the production of multiple virulence factors via intercellular communication. The QS process is triggered by an auto-inducer (AI), a diffusible molecule initially generated at a low concentration [14]. As cell density escalates, the AI concentration steadily elevates until it attains a critical threshold level. Subsequently, the AI re-enters the bacteria and selectively attaches to its designated target protein to form an AI–protein complex [15]. The AI–protein complex stimulates the activation of genes responsible for regulating several virulence factors and the formation of biofilm [16]. Hence, an effective approach to control biofilm formation is to disrupt this communication system using specific natural substances, with plant-based compounds emerging as the most promising alternatives. Because of their wide variety and intricate biochemistry, natural substances exhibit a broad array of mechanisms against QS mediators, effectively impeding the formation of biofilms.

A recent study has unveiled that numerous natural substances exert significant effects on QS mediators [17,18]. Medicinal plants have shown a strong propensity for hindering the development of biofilms [19]. EOs, present in various medicinal plants, have garnered substantial attention in the field of medical chemistry [20]. The intricate composition of EOs enables them to modulate various biological processes through multiple mechanisms of action. It plays a crucial role in inhibiting bacterial communication by demonstrating its anti-quorum sensing (anti-QS) capabilities [21].

*Matricaria aurea (M. aurea)*, often known as Golden Chamomile, is a widely used medicinal herb in Jordan. It is a member of the Asteraceae family. It is dispersed over Europe and the Mediterranean region. *M. aurea* and *Matricaria chamomilla* are distinct species, both referred to as babunij, and are utilized in traditional medicine as unified herbal remedies. *M. aurea* is often grown in the southern region of Jordan, while *Matricaria chamomilla* grows in the northern region of Jordan. The flowers of *Matricaria* sp. are commonly employed in traditional medicine to alleviate the symptoms of gastroenteritis, including stomach ache, abdominal pain, and diarrhea. Additionally, they are utilized to cure and alleviate respiratory tract symptoms such as sore throat, bronchitis, cough, and cold [22]. Nevertheless, *M. aurea* has received limited scientific attention. Some research has demonstrated the analgesic, anti-inflammatory, antibacterial, and antioxidant properties of extracts derived from this plant [23,24]. In their study, Kheder et al. [24] discovered that the EO derived from Tunisian *M. aurea* exhibits noteworthy antibacterial properties against *Staphylococcus aureus*, *Micrococcus luteus*, and *Escherichia coli*. This was observed through the low values of the minimum inhibitory concentration (MIC), which ranged from 50 to 100 µg/mL. However, when tested against *P. aeruginosa*, the EO did not show any inhibitory effect even at the highest concentration tested (1 mg/mL).

As a result, *M. aurea* was chosen for this study based on its historical traditional use and its modest antibacterial activity against *P. aeruginosa*. The primary aim of this study was to assess the influence of the *M. aurea* EO on the QS system and biofilm formation in *P. aeruginosa*. An examination was carried out to evaluate the effect of QS-related genes, specifically *phaZa1*, *aprA, pelA,* and *pslA*, on their gene expression. Furthermore, the chemical composition of the *M. aurea* EOs was scrutinized using gas chromatography-mass spectrometry (GCMS).

## 2. Results

### 2.1. Chemical Composition of M. aurea EO

A pale-yellow EO was extracted from the aerial parts of *M. aurea*. The average yield of the oil was 0.21% (*v*/*w*) based on the dry weight of the plant. The GCMS analysis showed the presence of 35 components, which accounted for approximately 95.8% of the EO from *M. aurea*. The primary constituents of this EO, as indicated in Table 1, consisted of oxygenated sesquiterpenes (66.8%) and hydrocarbon sesquiterpenes (17.5%). The chemical α-bisabolol oxide A accounted for the highest proportion at 64.8%, followed by β-bisabolene at 6.3% and α-farnesene at 4.8%.

The components are recorded based on their elution order from a DB5-SMS column. KI_cal_ refers to the computed KI concerning a combination of n-alkanes (C8–C31) on a DB5-SMS column. KI_let_: KI values of the detected compounds as documented in the NIST library.

### 2.2. Antibacterial Activity

The inhibition zone for the positive control, erythromycin, was 10.33 mm, while there was no inhibition zone observed for the negative control dimethyl sulfoxide (DMSO) (Table 2). According to the inhibition zone (12.8 mm) and MIC value (1.25 mg/mL), the *M. aurea* EO showed moderate antibacterial activity against *P. aeruginosa*.

### 2.3. Cell Viability

**Figure 1:**
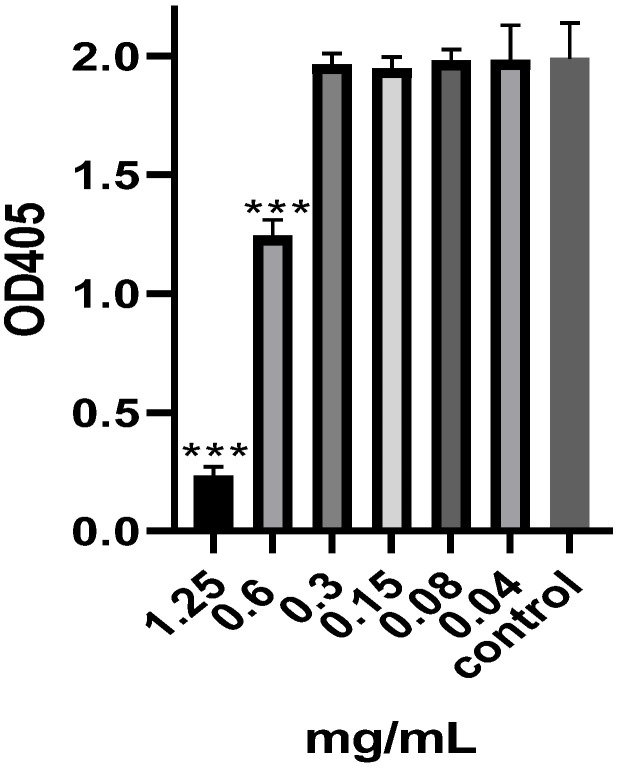
Effect of the different concentrations of *M. aurea* EO on the viable *P. aeruginosa* cells as measured by the absorbance of the red color resulting from formazan production. *** *p* ˂ 0.001 compared to control untreated cells.

The assay was performed to confirm that the EO’s antibiofilm activity was not due to a reduction in viable cells (Figure 1). A significant reduction (*p* < 0.001) in the viable cell count was observed at concentrations equal to 1.25 mg/mL (MIC) and 0.6 mg/mL (½ MIC). Cell viability was not significantly reduced at lower concentrations. DMSO, the negative control, exhibited no effect on *P. aeruginosa* viability.

### 2.4. Antibiofilm

**Figure 2:**
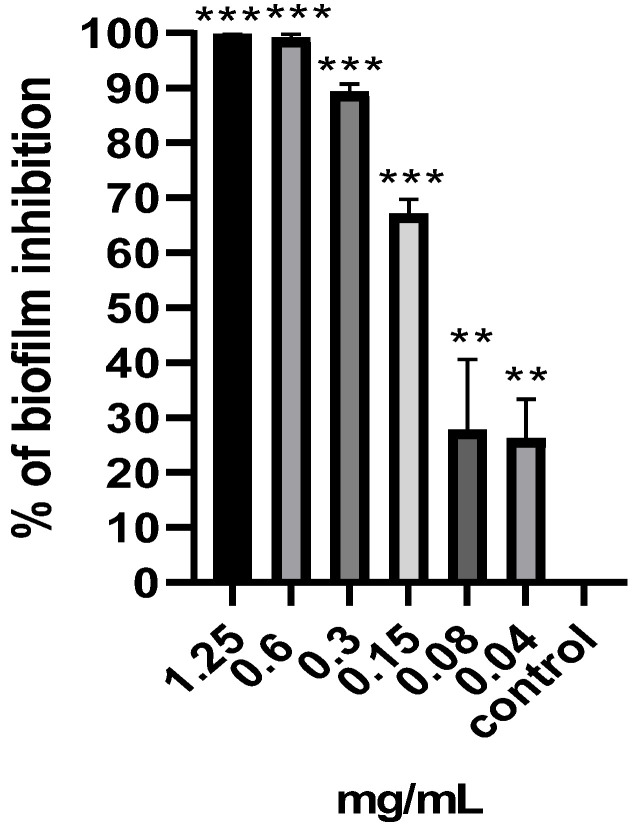
Percentage of biofilm inhibition of *P. aeruginosa* after treated with various concentrations of *M. aurea* EO, as determined by the crystal violet assay. ** *p* ˂ 0.01 and *** *p* ˂ 0.001 compared to control untreated cells.

The effect of the *M. aurea* EO on the capability of *P. aeruginosa* to develop a biofilm was evaluated using various concentrations (0.04–1.25 mg/mL) (Figure 2). A complete inhibition of the *P. aeruginosa* biofilm (percent of inhibition greater than 90%) was achieved at treatment concentrations equal to MIC (1.25 mg/mL), ½ MIC (0.6 mg/mL), and ¼ MIC (0.3 mg/mL). However, the MBIC50 appears to be within the range between 0.15 and 0.08 mg/mL. Hence, the suppression of biofilm development at doses of 1.25 and 0.6 mg/mL was due to the decrease in viable cells. Consequently, the concentrations of 0.3, 0.15, and 0.08 mg/mL have been selected for further analysis since they have no significant effect on cell viability. DMSO, the negative control, exhibited no antibiofilm activity.

### 2.5. Biofilm Visualization

**Figure 3:**
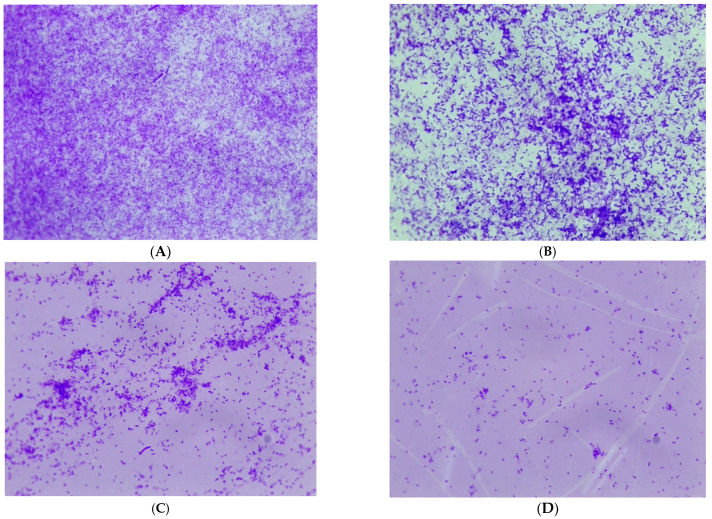
Images of a light microscope (40×) observation for the ability of *P. aeruginosa* to form biofilm after being treated with *M. aurea* EO at different concentrations equal to (**A**): 0.0, (**B**): 0.08, (**C**): 0.15, and (**D**): 0.3 mg/mL.

The effect of the *M. aurea* EO on the biofilm of *P. aeruginosa* was observed using a light microscope and SEM. The light microscope image revealed the presence of densely aggregated cells in the untreated *P. aeruginosa* biofilm (Figure 3A). After being treated with the *M. aurea* EO, the density of the aggregated cells dropped and became more dispersed, reaching its peak at a concentration of 0.3 mg/mL (Figure 3B–D).

**Figure 4:**
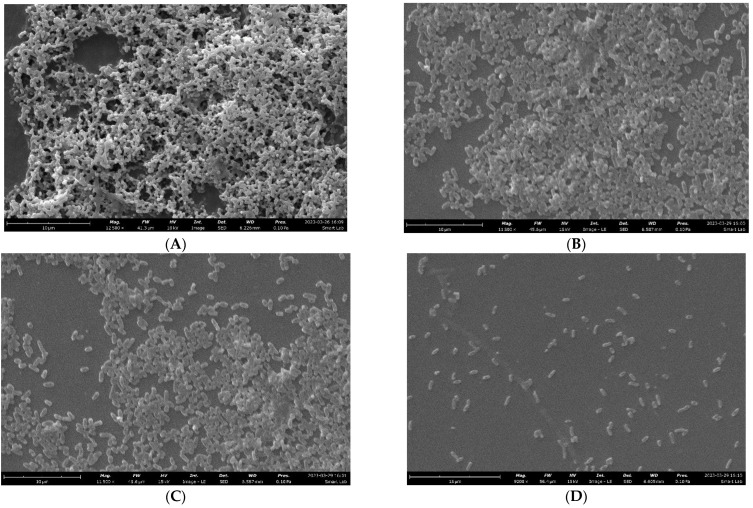
Images of SEM observation for the ability of *P. aeruginosa* to form biofilm after being treated with *M. aurea* EO at different concentrations equal to (**A**): 0.0, (**B**): 0.08, (**C**): 0.15, and (**D**): 0.3 mg/mL.

The effect was also studied using SEM. The picture illustrates the typical structure of a *P. aeruginosa* biofilm, which consists of multiple layers of clustered cells (Figure 4A). This biofilm can be characterized as an architectural arrangement with interconnected channels. Conversely, the *P. aeruginosa* biofilm that was treated with the *M. aurea* EO exhibited dispersed and non-adherent cells, with these indications becoming more pronounced at a higher concentration (0.3 mg/mL) (Figure 4B–D). These findings suggest that the treatment with the *M. aurea* EO may lead to a decrease in the capacity of *P. aeruginosa* to produce a biofilm.

### 2.6. Violacein Inhibition of C. violaceum

**Figure 5:**
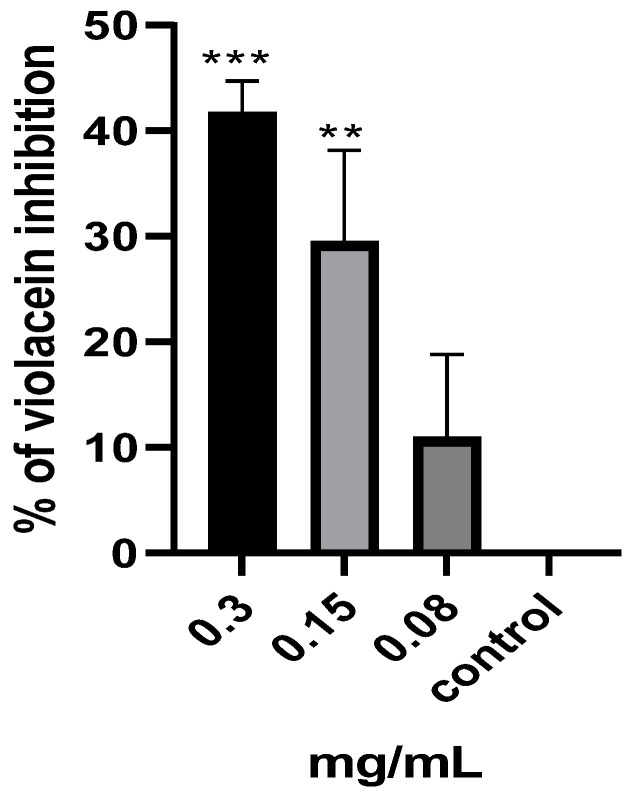
Percentage of violacein inhibition production by *C. violaceum* treated with various concentrations of *M. aurea* EO. The percentage of violacein inhibition for the untreated culture (control) was considered 0.0%. ** *p* ˂ 0.01 and *** *p* ˂ 0.001 compared to control untreated cells.

The impact of the *M. aurea* EO on the QS system of *C. violaceum* was initially assessed using the well diffusion method. The findings indicated that the well containing 1 mg of the EO resulted in a 13 mm zone of inhibition in violacein production. A quantitative investigation was conducted to determine the production of violacein, and the percentage of violacein inhibition was obtained (Figure 5). There was a significant decrease in violacein production when the treatment concentrations were 0.3 (*p* < 0.001) and 0.15 (*p* < 0.01) mg/mL, resulting in a respective inhibition of 41.68 and 29.47%. The results suggest that the *M. aurea* EO exhibits significant anti-QS activity.

### 2.7. Effect on Biofilm Development

#### 2.7.1. Swarming Motility

**Figure 6:**
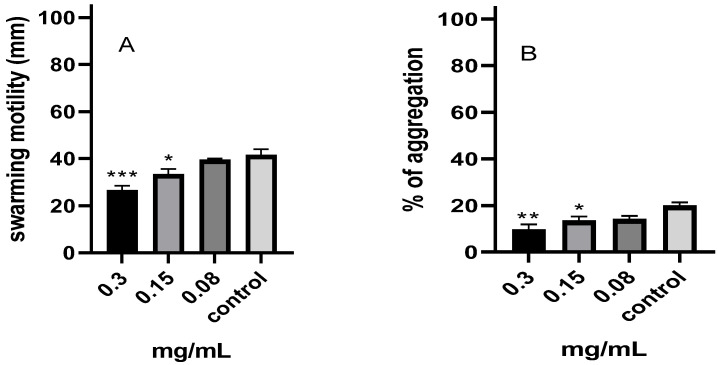

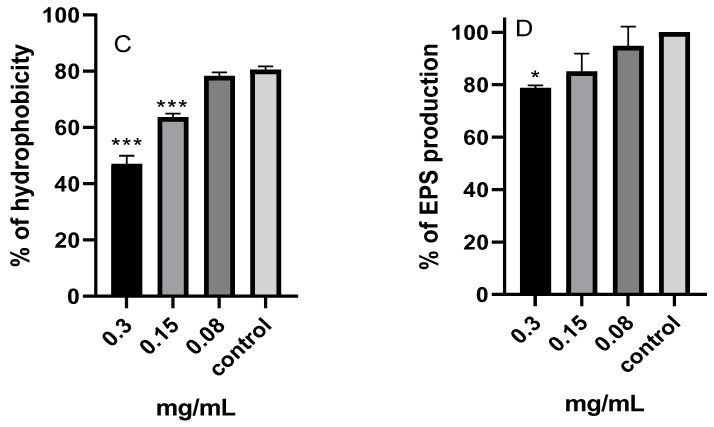
Effect of the different concentrations of *M. aurea* EO on the swarming motility (**A**), aggregation (**B**), hydrophobicity (**C**), and EPS production (**D**) of *P. aeruginosa*. * *p* ˂ 0.05, ** *p* ˂ 0.01 and *** *p* ˂ 0.001 compared to control untreated cells.

Figure 6A demonstrates that the untreated *P. aeruginosa* had a swarming motility of 41.5 mm. When *P. aeruginosa* was treated with the *M. aurea* EO, a decrease in its motility was found, and this decrease was dependent on the dosage. The swarming motility diameter was significantly decreased to 26.5 (*p* < 0.001) mm and 39.5 (*p* < 0.05) mm at dosages of 0.3 mg/mL and 0.15 mg/mL, respectively. The swarming motility was not significantly reduced by the treatment with 0.08 mg/mL.

#### 2.7.2. Aggregation

When the untreated cells were compared to the cells treated with 0.3 mg/mL of the *M. aurea* EO, a substantial decrease in the aggregation ability of *P. aeruginosa* was seen (Figure 6B). The untreated cells had an aggregation percentage of 19.84%, while the treated cells had a reduced aggregation percentage of 9.48% (*p* ˂ 0.01) (Figure 6B). The treatment with a concentration of 0.15 mg/mL resulted in a statistically significant reduction (*p* ˂ 0.05) in the aggregation ability, reducing it to 13.43%. There was no notable decrease in the ability to form aggregates at a concentration of 0.08 mg/mL.

#### 2.7.3. Hydrophobicity

The data demonstrated that the application of the *M. aurea* EO resulted in a significant (*p* ˂ 0.001) decrease in the hydrophobicity of *P. aeruginosa* when treated with concentrations of 0.3 and 0.15 mg/mL (Figure 6C). The hydrophobicity exhibited a substantial decrease from 80.52% (untreated) to 46.98 and 63.64%, respectively. The hydrophobicity did not significantly decrease after treatment with 0.08 mg/mL of the *M. aurea* EO.

#### 2.7.4. EPS Production

When the *P. aeruginosa* cells were treated with 0.3 mg/mL of the *M. aurea* EO, there was a significant reduction (*p* ˂ 0.05) in EPS generation to 78.73% compared to the untreated cells (Figure 6D). Nevertheless, the administration of 0.15 and 0.08 mg/mL leads to a decrease in the EPS, although this decrease is not statistically significant.

### 2.8. Virulence Factors

#### 2.8.1. Pyocyanin

**Figure 7:**
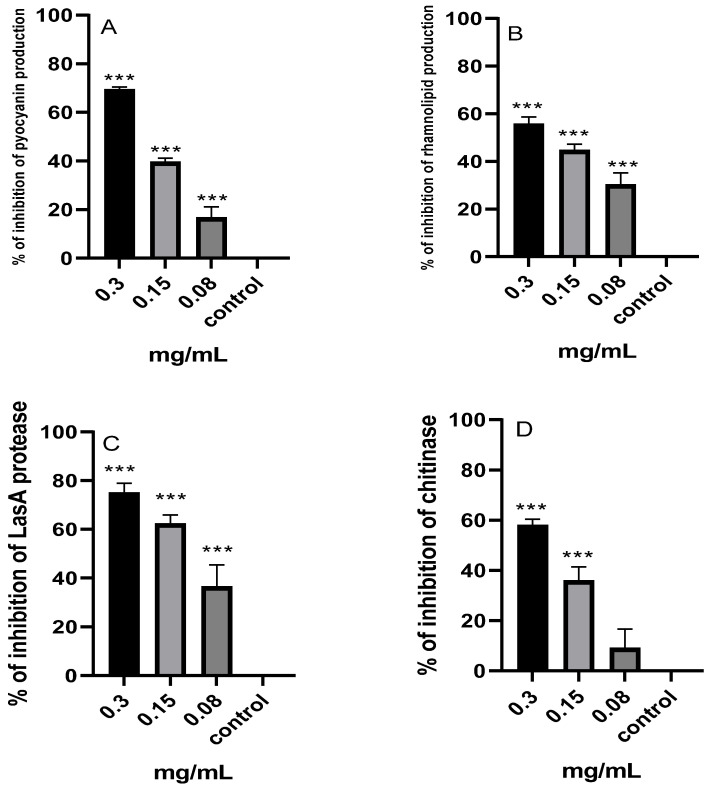
Percentage inhibition of pyocyanin (**A**) and rhamnolipids (**B**) production, and LasA protease (**C**) chitinase (**D**) activity by *P. aeruginosa* treated with various concentrations of *M. aurea* EO. The percentage of inhibition for the untreated *P. aeruginosa* (control) was considered 0.0%, *** *p* ˂ 0.001 compared to control untreated cells.

The effect of the *M. aurea* EO on the production of pyocyanin in *P. aeruginosa* was evaluated (Figure 7A). A dose-dependent inhibition of pyocyanin production was observed. A significant (*p* ˂ 0.001) reduction in pyocyanin production at the treatment concentrations of 0.3, 0.15, and 0.08 mg/mL resulted in a percentage of pyocyanin inhibition of 69.44, 39.69, and 16.7%, respectively.

#### 2.8.2. Rhamnolipids

As depicted in Figure 7B, the evaluation was conducted to determine the impact of the *M. aurea* EO on the synthesis of rhamnolipids in *P. aeruginosa*. A suppression of rhamnolipids synthesis was detected, which varied depending on the dose. There was a statistically significant decrease (*p* ˂ 0.001) in the formation of rhamnolipids at treatment concentrations of 0.3, 0.15, and 0.08 mg/mL. This led to a percentage inhibition of rhamnolipids production of 55.69, 44.68, and 30.29%, respectively.

#### 2.8.3. LasA Protease

The impact of the *M. aurea* EO on the activity of LasA protease in *P. aeruginosa* was assessed (Figure 7C). A decrease in LasA protease activity was observed, with the extent of the decrease varying depending on the concentration. Protease activity exhibited a statistically significant reduction (*p* ˂ 0.001) at treatment concentrations of 0.3, 0.15, and 0.08 mg/mL, resulting in corresponding inhibition percentages of 75.08, 62.39, and 36.52%.

#### 2.8.4. Chitinase Activity

An evaluation was conducted to investigate the influence of the *M. aurea* EO on the activity of chitinase in *P. aeruginosa*, as shown in Figure 7D. An observed reduction in chitinase activity was noted, with the percentage of the reduction varying according to the concentration. The chitinase activity showed a significant decrease (*p* ˂ 0.001) when treated with doses of 0.3 and 0.15 mg/mL, resulting in inhibition percentages of 58.12 and 36.05%, respectively. There was no substantial decrease observed at the treatment concentration of 0.08 mg/mL.

### 2.9. Mechanism of QS

#### 2.9.1. N-Acyl Homoserine Lactones (AHL)

**Figure 8:**
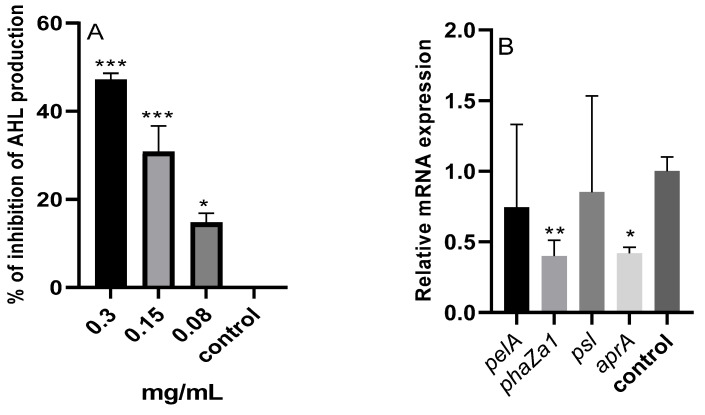
The percentage of inhibition of AHL production (**A**) and the relative mRNA expression of *pelA*, *phaZa1*, *psl*, and *aprA* genes (**B**) of *P. aeruginosa* treated with various concentrations of *M. aurea* EO. * *p* ˂ 0.05, ** *p* ˂ 0.01 and *** *p* ˂ 0.001 compared to control untreated cells.

As seen in Figure 8A, the assessment was carried out to ascertain the impact of the *M. aurea* EO on *P. aeruginosa’s* production of AHL. There was a discernible drop in AHL, with the percentage of the inhibition changing according to the concentration. At doses of 0.3 and 0.15 mg/mL, the generation of AHL decreased significantly (*p* < 0.001), with corresponding inhibition percentages of 47.14 and 30.76%. Significant (*p* < 0.05) was also the notable drop of 14.74% at the treatment concentration of 0.08 mg/mL.

#### 2.9.2. qRT-PCR

The mRNA expression levels of four genes were studied using RT-PCR (Figure 8B). Treating *P. aeruginosa* with a concentration of 0.3 mg/mL of the *M. aurea* EO resulted in a significant reduction in the expression of *phaZa1* (*p* < 0.01) and *aprA* (*p* < 0.05) genes. There was no significant reduction in the expression of the *pelA* and *psl* genes.

## 3. Discussion

Biofilms present a substantial and enduring threat, especially within the realm of various diseases, as they are linked to elevated rates of both illness and mortality [25,26]. Biofilms frequently exhibit limited susceptibility to antibiotics, prompting the need for the exploration and development of alternative methods to address infectious diseases associated with biofilms [27]. QS is a crucial mechanism that regulates the expression of genes associated with virulence factors. This regulatory system functions by assessing cell density and relies on extracellular signals [27,28]. Consequently, the substances that specifically target the QS system will ultimately hinder intercellular communication by exerting an influence on the genes that are engaged in QS. Medicinal herbs are rich in natural compounds that possess the ability to interfere with QS signals, consequently exerting a direct impact on the control of virulence factors linked to a wide range of diseases [18,29,30].

The current work utilized the EO of *M. aurea* to target *P. aeruginosa* and analyzed its efficacy against different virulence factors expressed by this bacterium. It showed that α-bisabolol oxide A, β-bisabolene, and α-farnesene were the main ingredients in the *M. aurea* EO. Few investigations have documented the chemical composition of *M. aurea*, revealing the existence of various significant chemicals that may be attributed to both abiotic and biotic causes. Khan et al. [31] found that sesquiterpene hydrocarbons were the main chemical class present in the aerial parts of Jordanian *M. aurea* oil. Among these, (E, E)-α-farnesene accounted for 50.2% of the composition, followed by γ-gurjunenepoxide at 8.5%, (E)-β-farnesene at 8.1%, and (Z, E)-α-farnesene at 4.4%. On the other hand, the most dominant chemical group in the Saudi *M. aurea* EO was oxygenated sesquiterpenes. The dominant compositions in this class were α-bisabolol at 27.8%, γ-gurjunenepoxide at 21.7%, (E, E)-α-farnesene at 16.3%, and cis-spiroether at 7.5%. The primary components of the *M. aurea* EO, as described in another study from Saudi Arabia, are bisabolol oxide A (64.8%), n-Nonadecane (6.7%), and (2R,3R, ALL-E)-2,3-epoxy-2,6,10,14-tetramethyl-16-(phenylthio) hexadeca-6,10,14-triene (5.8%) [32]. A prior study conducted in Iran indicated that α-bisabolene oxide A (59%), α-bisabolol oxide A (22.3%), and chamazolene (8.8%) were the primary constituents of the *M. aurea* EO [33]. When comparing the current study with existing research, it became evident that the EO from *M. aurea* contained similar chemical components, but their respective proportions varied significantly. The differences in the EO composition can be credited to many factors, such as weather conditions, nutrient availability, soil type, and the timing of harvesting.

The results of the antibacterial activity of the *M. aurea* EO against *P. aeruginosa* are in agreement with other reports [24,34]. Studies also showed that the antibacterial activity of the methanol, ethanol, acetone, ethyl acetate, and chloroform extracts of *M. aurea* was mild against *P. aeruginosa* [35]. However, the *M. aurea* EO and extracts possess broad-spectrum antibacterial activity against both gram-positive and gram-negative bacteria [24,34,35]. In addition, plant species belonging to the same family, Asteraceae, have been found to exhibit broad-spectrum antimicrobial activity against Gram-positive and Gram-negative as well as *P. aeruginosa*, such as *Ageratum conyzoides*, *Tagetes erecta*, *Eucalyptus occidentalis*, *E. striaticalyx*, and *E. stricklandii* [36,37,38].

The results of this study also underscored that the EO from *M. aurea* possesses the capability to inhibit biofilm formation without impacting the viability of the cells. Prior research has yielded similar outcomes, demonstrating that EOs derived from different plants effectively hinder the production of biofilms by various microbes. For instance, reports on the EOs from *Tagetes minuta*, cinnamon oil, *Thymus daenensis*, and *Satureja hortensis* found that they effectively prevented the production of biofilms, as well as other virulence factors mediated by QS [31,39,40]. Plants belonging to the same family as *M. aurea* have been reported to have antibiofilm activity, such as *Launaea capitata* [41], *Helichrysum caespitium* [42], and *Vernonia adoensis* [43].

The biofilm develops in a series of stages, starting with the creation of the initial biofilm layer, then followed by the movement of microorganisms to the surface, attachment, reproduction, maturation, and finally detachment [44]. The initial stage of biofilm growth was determined to be primary attachment and surface adherence. Bacterial adherence is primarily facilitated by the extracellular polymeric matrix composition, cell surface hydrophobicity, and flagellar motility [45]. This study found that the EO of *M. aurea* effectively reduced the hydrophobicity of bacteria. The decrease in hydrophobicity can be attributed to the presence of EO components that bind to the site of adhesion, thereby diminishing the hydrophobicity of the bacteria. The hydrophobicity of a surface affects the early attachment phase in the formation of a biofilm. The reduction in hydrophobicity may account for the decrease in biofilm growth by inhibiting adhesion. Furthermore, the results indicated that the *M. aurea* EO hindered bacterial aggregation, hence impeding the formation of biofilms. Furthermore, bacterial motility is a highly significant component that can affect the onset of biofilm development. Swarming motion substantially facilitates the first adhesion [46]. A crucial element necessary for *P. aeruginosa* swarming movement is the synthesis of rhamnolipid as a biosurfactant, which functions to lower surface tension and enable motility across the surface [47]. This study found that the sub-MIC *M. aurea* EO had a dose-dependent inhibitory effect on swarming motility and rhamnolipids production. This may suggest that one of the mechanisms preventing swarming motility is the inhibition of rhamnolipid production. O’May and Tufenkji [48] reported that a rhamnolipid supplement could partially restore the inhibition of swarming motility caused by the extracts.

The production of EPS is necessary for the establishment of the biofilm structure and the formation of the microcolony. The presence of EPS in bacteria provides a protective barrier that hinders the entry of antibiotics into the bacterial cell, hence contributing to antibiotic resistance [49]. Furthermore, the formation of EPS leads to alterations in the structure of biofilms, which are linked to heightened resistance against antibacterial medications. Therefore, decreasing the formation of EPS will enhance the elimination of biofilms by intensifying their contact with antimicrobial substances. This study demonstrates that the *M. aurea* EO significantly reduces the production of EPS.

A preliminary investigation showed that *M. aurea* EO possesses anti-QS activity, as indicated by the reduction in violacein production in *C. violaceum*. Violacein is produced via *C. violaceum* as an acyl homoserin lactone purple pigment. The inhibition of this pigment production indicates an inhibition of the QS pathway and an agent that inhibits the production of violacein was considered a possible anti quorum-sensing candidate [50].

QS is a crucial regulatory mechanism employed by bacteria, including the opportunistic pathogen *P. aeruginosa*, to coordinate gene expression in a population-dependent manner [51]. *P. aeruginosa* utilizes QS-dependent genes to sense and respond to changes in cell density through the production and detection of signaling molecules, such as N-acyl homoserine lactones [51]. The activation of QS-dependent genes allows *P. aeruginosa* to synchronize behaviors, such as biofilm formation, virulence factor secretion, and antibiotic resistance, in a concerted manner [52]. This sophisticated communication system enhances the adaptability and survival of the bacterial community in diverse environments. Understanding the role of QS-dependent genes is crucial for devising strategies to disrupt bacterial communication and control pathogenicity [52].

The use of *M. aurea* resulted in a substantial decrease in the production of protease, chitinase, pyocyanin, and rhamnolipid by *P. aeruginosa*, as seen in this investigation. The production of these molecules occurs outside of cells, and they serve as markers for assessing the QS system [53]. These variables have a crucial role in the pathogenicity of *P. aeruginosa*. The secretion of elastase and protease plays a crucial role in adhesion and colonization. Pyocyanin oxidizes glutathione and reduces its concentration in airway epithelial cells, hence diminishing an essential cellular antioxidant that plays a crucial role in regulating redox-sensitive signaling pathways [54,55]. Rhamnolipids function as a surfactant and facilitate the initiation of biofilm formation [56]. The substantial decrease in the generation of these harmful substances suggests that the *M. aurea* EO exhibits unique anti-QS activity that may disrupt the ability of *P. aeruginosa* to cause disease and impede the progression of the infection.

Administering *M. aurea* to *P. aeruginosa* led to a notable decrease in the release of AHL. This finding suggests that the EO hindered QS by interfering with the QS mechanisms, such as the las, rhl, and pqs systems [57]. Inhibiting these QS systems decreases the production of autoinducers (AIs) such as AHL. *P. aeruginosa* has a minimum of four operational QS systems, with two of them controlled by an AHL called Las and Rhl. The other two systems are regulated by quinolones and carbaldehydes, known as the Pqs and Iqs QS systems, respectively. The LAS system regulates the Rhl system, while the PQS system up-regulates the Rhl circuit. The Iqs system governs the Pqs system and RhlA system [58].

AIs control the activation of virulence pathways, enhance their signaling route through positive feedback, and have the ability to modify other systems through interconnected QS circuits [59]. The activation of these QS circuits controls the gene expression of different virulence factors. The Las circuit induces the activation of genes such as *lasB* and *lasA* (elastases A and B), *aprA* (AprA alkaline protease), as well as *psl* and *pel* (Psl and Pel exopolysaccharides) [60]. Furthermore, the Rhl circuit not only controls these virulence pathways but also stimulates the release of rhamnolipids [61,62]. The Pqs circuit primarily induces the release of pyocyanin, as well as the synthesis of elastases A and B, hydrogen cyanide, lectins A and B, two types of exopolysaccharides (Pel and Psl), and rhamnolipids [63,64]. This study also assessed the impact of *M. aurea* on the mRNA expression of the las-dependent genes *aprA*, *psl*, and *pel*, as well as the rhl-dependent gene *phaZa1*. The activation of the *phaZa1* gene regulates the release of pyocyanin at a high level [65]. *P. aeruginosa* releases the virulence factor alkaline protease (AprA) to improve its ability to survive. AprA enzymatically cleaves monomeric flagellin, resulting in reduced activation of Toll-like receptor 5. Furthermore, AprA enzymatically breaks down host proteins, including complement proteins and cytokines. According to reports, QS has a considerable impact on the expression of the Pel and Psl operons. These operons play a crucial role in the production of two important matrix polysaccharides, Pel and Psl [66]. The secretion of these chemicals is essential for the processes of biofilm adherence, structure, and protection. Treating *P. aeruginosa* with *M. aurea* resulted in a significant decrease in the expression of *aprA* and *phaZa1* genes. However, there was no significant difference in the expression of the *pelA* and *pls* genes. *P. aeruginosa*, in reality, generates a minimum of three distinct polysaccharides outside of its cells, which contribute to the formation of biofilms. These polysaccharides are known as alginate, PEL, and PSL [67]. *P. aeruginosa* organizes the production of its biofilm matrix exopolysaccharides. *P. aeruginosa* decreases the production of other polysaccharides when the production of one specific polysaccharide is raised to preserve equilibrium in the general quantity of exopolysaccharides. The excessive production of Psl significantly reduced the synthesis of alginate. The activation of Pel also inhibited the production of Psl polysaccharides. Additionally, the excessive production of alginate had a significant impact on the synthesis of other lipopolysaccharides [68].

More than 5000 sesquiterpenes derivatives have been identified, and the Asteraceae family appears to be the major supplier of these derivatives. They have been reported to have broad biological activity, including antimicrobial, anti-tumor, anti-ulcer, and anti-diabetic activities [69]. The biofilm and QS inhibitory effect of the *M. aurea* EO can be related to the presence of its primary components of sesquiterpenes, including α-bisabolol oxide A, β-bisabolene, and α-farnesene. However, the combined effect of both main and minor compounds should not be ignored. Significantly, extracts containing a high concentration of bisabolol and bisabolol derivatives have been found to possess antibiofilm and anti-QS properties. The concentration of 0.5 µg/mL of *Matricaria chamomilla’s* EO, which consists of α-bisabolol oxide B (7.3%) and α-bisabolol (7.3%) as its main constituents, leads to a notable decrease in both *P. aeruginosa* biofilm and alginate formation [70]. The extract from the brown macroalga *Padina gymnospora*, which contains 69% α-bisabolol, reduces the formation of biofilms and the expression of virulence factors controlled by QS in *Serratia marcescens* [71].

Certain limitations need to be emphasized in this study. The investigation was carried out using a single clinically isolated bacterium, potentially impeding the broad applicability of this study’s outcomes. *P*. *aeruginosa* serves as the model organism, and to enhance result generalization, the inclusion of other bacteria, such as *S. aureus* and *E. coli*, is necessary. To ensure the trial’s quality, positive controls are essential for both biofilm and virulence factors. Additional parameters are requisite to validate the inhibitory impact on biofilm and virulence factors and substantiate the findings. The examination of only a few quorum-sensing genes has taken place, and incorporating other genes or employing more sophisticated protocols could contribute to elucidating the precise mechanism of action.

## 4. Materials and Methods

### 4.1. Chemicals

Tetrazolium salt 2,3,5-triphenyl-tetrazolium chloride (TTC), oricinaol, and Carbazole were from Santa Cruz Biotechnology, Dallas, TX, USA. Azocasein and n-hexadecane were purchased from Sigma Aldrich, St. Louis, MO, USA. Trichloroacetic acid (TCA) was from Thermo Fischer Scientific, Shanghai, China. Chitin azure was from Bioscientific Carbosynth, Compton, UK. Muller Hinton broth (MHB), Muller Hinton Agar (MHA), and Luria Bertani broth (LB) were from Eco biolab, Budapest, Hungary. DMSO was from Alpha Chemika, Mumbai, India. Erythromycin disc was from Oxoid, UK. Diethyl ether was from SDFCL, Mumbai, India. Ethanol and ethyl acetate were from AZ chem, EU, Phoenix, AZ, USA.

### 4.2. Plant Materials and EO Extraction

*M. aurea* was collected in March 2021 from the Al-Karak region in Jordan. Dr. Feryal Al-Khresat from the Department of Biology at Mu’tah University in Al-Karak, Jordan successfully determined the taxonomic classification of the plant. The voucher specimens (NO: MU2023-08) were stored in the Department of Biology, Faculty of Science, Mutah University, Mutah, Jordan.

A 100 g sample of the plant material’s fresh aerial parts was subjected to hydro-distillation for 3 h utilizing simple Clevenger equipment. The aforementioned technique was repeated more than 10 times. The oil was separated from the water phase (1 L) using diethyl ether (100 mL). The diethyl ether was volatilized, and the oil was dehydrated using anhydrous sodium sulfate. Ultimately, the obtained oil was preserved at a temperature of 4 °C until it could be further examined.

Stock solutions of the *M. aurea* EO were made using 10% DMSO. For treated cultures, the required final concentrations of the oil were prepared using sterile broth, and the adjusted bacterial culture was added in a proportion of 1:20 (*v*:*v*). The untreated culture was prepared using broth media and the adjusted bacterial culture in a proportion of 1:20 (*v*:*v*). In all experiments, a solution containing broth media, the extract solvent (DMSO), and the adjusted bacterial culture in a proportion of 1:20 (*v*:*v*) was used as a negative control.

### 4.3. Gas Chromatography Mass Spectrometry

The GCMS investigation was performed using a Shimadzu qp2010 Plus (Kyoto, Japan). A column of DB5-SMS (30 m × 0.25 mm ID × 0.25 m of film thickness) was utilized. The volume of injection was 1.0 in a ratio of 1:100. Helium was used as a mobile phase, and it was operated at a flow rate of 1 mL/min. The heating rate was programmed at 4 °C/min starting from 50 °C (initial temperature) to 290 °C (final temperature) and held at 50 °C for five minutes with a total run time of 68 min. Both the injector and transfer-line temperatures were set at 250 °C. Electron ionization was the mode used in the MS with a 70 electron volt (eV) electron energy and a 250° C ion source temperature. The identification of the eluted peaks was made based on the retention time and the linear index of standard alkane compounds (C8–C20). In addition, the MS spectra of the candidate compounds were compared with their analogs in the NIST library and published data [24,31,72]. Indeed, co-chromatography was performed for certain compounds (α-terpinene, limonene, β-caryophyllene, α-humulene, and myristicin) under similar circumstances. The percentage of each identified compound was calculated based on the peak area of the identified compound as a proportion of the total area of all detected peaks.

### 4.4. Bacterial Strains

*P. aeruginosa* was obtained from a urine sample of a patient diagnosed with a urinary tract infection at the AlKarak Government Hospital in Karak, Jordan. The identification of the bacteria was performed using the Biomérieux VITEK^®^ 2 system, Marcy-l’Étoile, France. The organism was identified as *P. aeruginosa,* which produces β-Lactamase. The strain *Chromobacterium violaceum* ATCC 12472 was acquired from the American Type Culture Collection (ATCC, Manassas, VA, USA).

The preparation of the bacterial culture was made by inoculating one single colony into a sterile 5 mL nutrient broth (NB) and incubating for 24 h at 37 °C. The prepared culture was adjusted to 0.5 McFarland Standard (1.5 × 10^8^ CFU/mL) using sterile broth.

### 4.5. Antibacterial Activity

#### 4.5.1. Disc Diffusion Assay

The experiment was conducted using a cell density of 1.5 × 10^8^ of *P. aeruginosa* with MHA as the medium. A volume of 100 µL from a 24 h old bacterial culture was applied onto the agar surface using a swab. Subsequently, an azsterile disc was loaded with 1 mg of the prepared EO. The disc was allowed to dry and thereafter put onto the surface of the agar plate. Erythromycin (10 µg) was also tested as a positive control. The inhibitory zone that formed after the 24 h incubation at 37 °C was measured in millimeters [73].

#### 4.5.2. MIC

The minimum inhibitory concentration (MIC) of the *M. aurea* EO against *P. aeruginosa* was assessed by utilizing a stock solution of 10 mg/mL of 10% DMSO. A 96-well plate was utilized, along with MHB (Mueller Hinton Broth). The EO was diluted by a factor of two to achieve final concentrations of 5, 2.5, 1.25, 0.6, 0.3, 0.15, 0.08, 0.04, 0.02, and 0.01 mg/mL. Then, a 10 µL bacterial inoculum containing a 1.5 × 10^8^ CFU/mL was added. The MIC is determined when the concentration being tested completely inhibits the observable growth of the bacteria after 24 h incubation at 37 °C using an ELIZA reader (MCL-2100C, Guangzhou, China) [74].

### 4.6. Cell Viability

A 96-well plate was prepared in a manner identical to the one prepared in the MIC methodology. After being incubated for 24 h at a temperature of 37 °C, the contents of each well were removed and cleaned extensively. Following the drying process, 200 µL of broth media containing 0.2% glucose and 50 µL of TTC (tetrazolium salt 2,3,5-triphenyl-tetrazolium chloride) solution were introduced into each well. The samples were then incubated in darkness at a temperature of 37 °C and a shaking speed of 150 rpm. The absorbance was measured at a wavelength of 405 nm after 6 h [27].

### 4.7. Antibiofilm

The antibiofilm efficacy was assessed using a crystal violet assay. A polystyrene 96-well plate was prepared in a manner identical to the one prepared in the MIC methodology. Following a 24 h incubation period at 37 °C, the contents of each well were discarded and thoroughly washed. Subsequently, crystal violet was introduced into each well. After 15 min of incubation at room temperature, the contents were removed and subsequently cleaned using tap water. Subsequently, an ethanol (98%) decolorizer was introduced into each well and left for 15 min. Next, the contents of each well were transferred to a new 96-well plate, and the optical density (OD590) was recorded. The biofilm inhibition percentage was determined relative to the optical density (OD590) of the standard *P. aeruginosa* biofilm [26].

### 4.8. Biofilm Visualization

Untreated and treated *P. aeruginosa* were cultivated on a 6-well plate with glass coverslips for 24 h at a temperature of 37 °C. Then, the coverslips were removed. A batch of these prepared coverslips was treated for inspection under a light microscope (NIKON, Tokyo, Japan). The coverslips were subjected to crystal violet staining for 1 min, followed by thorough rinsing and subsequent drying. The second set was utilized for scanning electron microscope (SEM) observation. The coverslips were fixed in 2.5% glutaraldehyde for 1 h. Furthermore, the coverslips were cleaned using a sodium acetate buffer with a concentration of 0.1 M and a pH of 7.3. Then, they were dehydrated using a sequence of ethanol solutions with different concentrations (10, 30, 50, 70, and 100%). Afterward, the samples underwent analysis using scanning electron microscopy (Thermo Scientific Phenom Desktop SEM, JU-24112022, Waltham, MA, USA).

### 4.9. Violacein Assay

The impact of the M. aurea EO on the synthesis of violacein by *C. violaceum* was assessed qualitatively by the well diffusion method and quantitatively using a spectrophotometric methodology. For the well diffusion approach, a volume of 100 µL of *C. violaceum* containing 1 × 10^6^ CFU/mL was evenly spread on LB agar. Subsequently, a well was created at the center of the cultivated plate, and 100 µL of DMSO containing 1 mg of the *M. aurea* EO was added to it. The incubation was conducted at a temperature of 37 °C for 24 h. The diameter of the inhibitory zone was determined in mm.

To conduct the quantitative study, untreated and treated cultures of *C. violaceum* were prepared and incubated at 37 °C for 24 h. Then, a 1 mL aliquot of each culture was subjected to centrifugation, and the resulting pellet was collected and suspended in DMSO. The mixture was agitated vigorously and the DMSO extract was subsequently separated from the cells using centrifugation. The concentration of crude violacein in the collected supernatant was quantified by measuring the absorbance at 575 nm with a comparison to the untreated control culture [75].

### 4.10. Effect on Biofilm Development

#### 4.10.1. Swarming Motility

The test was conducted using semisolid swarming agar plates [76]. Following autoclaving, varying amounts of the *M. aurea* EO were introduced into the empty Petri dishes, and subsequently, the autoclaved media was poured to achieve the desired final concentrations. The agar plates were allowed to solidify, and a single dot of *P. aeruginosa* was cultured. The motility zone was evaluated in millimeters after a 48 h incubation period at 37 °C.

#### 4.10.2. Aggregation

After the untreated and treated *P. aeruginosa* cultures were incubated for 24 h at 37 °C, the OD at 600 nm was measured using a spectrophotometer (Shimadzu UV-1601, Kyoto, Japan). Then, portions of these cultures were taken and vortexed for 60 s. Then, the OD was measured again. Accordingly, the percentage of aggregation was determined [77].

#### 4.10.3. Hydrophobicity

An initial absorbance at 600 nm (OD1) for untreated and treated cultures of P. aeruginosa incubated for 24 h at 37 °C was reported. Then, a 1:1 mixture of these cultures and n-hexadecane was made. The prepared mixture was vortexed using a vortex (Witeg, Wertheim, Germany) for 120 s. The aqueous phase was collected, and the OD at 600 nm was measured (OD2). Then, the percent of hydrophobicity was determined [78].

#### 4.10.4. EPS Production

The untreated and treated cultures of *P. aeruginosa* were incubated for 24 h at 37 °C. Subsequently, the culture underwent centrifugation, and a portion (1 mL) of the resultant supernatant was obtained and combined with cold ethanol (3 mL). Following a 24 h incubation at 4 °C, the combination underwent centrifugation, and the collected pellet was suspended in water (3 mL). To determine the total carbohydrates, the water containing EPS was treated with a 1:5 solution of phenol (5%) and H_2_SO_4_ (98%). The OD at 490 nm was reported, and the percentage of the EPS was determined about the untreated culture [79].

### 4.11. Effect on Virulence Factors

#### 4.11.1. Pyocyanin

The untreated and treated culture of *P. aeruginosa* was incubated for 24 h at 37 °C. Next, the culture was subjected to centrifugation (Bibby Sterilin Ltd., Bargoed, UK), and a part of the resulting supernatant was combined with chloroform to isolate the pyocyanin pigment. The extraction solution was vigorously agitated using a vortex mixer, and the resulting green-blue layer was isolated and combined with hydrochloric acid (HCl). The pink solution that was created was collected and the amount of pyocyanin present was quantified using a spectrophotometer at a wavelength of 520 nm [80].

#### 4.11.2. Rhamnolipids

The untreated and treated culture of *P. aeruginosa* was incubated for 24 h at 37 °C. Next, the culture was subjected to centrifugation, and a part of the resulting supernatant was combined with diethyl ether to isolate the rhamnolipids. Next, a fraction of the diethyl ether layer was collected, and the solvent was evaporated. The crude material was mixed with 200 µL of water. Subsequently, a solution of orcinol-sulfuric acid was introduced. The solution was placed in an incubator and maintained at a temperature of 24 °C for 30 min. The optical density at 421 nm was determined and used to compute the proportion of rhamnolipid formation [81].

#### 4.11.3. LasA Protease

The untreated and treated culture of *P. aeruginosa* was incubated for 24 h at 37 °C. Next, the culture was subjected to centrifugation, and a part (100 µL) of the resulting supernatant was collected. Then, 100 µL of azocasein and 3 mL of phosphate buffer (50 mM, pH 7) were introduced. The solution was incubated for 1 h at a temperature of 37 °C, with a shaking speed of 150 rpm. Subsequently, 500 microliters of trichloroacetic acid were introduced. Following a 10 min period, the solution underwent centrifugation, and the OD of the liquid portion was measured at 366 nm [82].

#### 4.11.4. Chitinase

An exact quantity of 1.3 mg/mL of chitin azure was introduced into 130 mL of a sodium phosphate buffer with a concentration of 50 mM (pH 7.0). The mixture was then incubated for 7 days at a temperature of 37 °C, with a shaking speed of 150 rpm. Simultaneously, both the untreated and treated culture of *P. aeruginosa* was incubated for 24 h at a temperature of 37 °C. Subsequently, the culture underwent centrifugation, and a portion (500 µL) of the resultant supernatant was obtained and combined with the chitin azure solution (4.5 mL). Following 24 h, the combination underwent centrifugation, and the OD of the resulting supernatant was reported at 570 nm [80].

### 4.12. Mechanism of QS

#### 4.12.1. N-Acyl Homoserine Lactones (AHL)

The untreated and treated cultures of *P. aeruginosa* were incubated for 24 h at 37 °C. Subsequently, the culture underwent centrifugation, and a portion (2 mL) of the supernatant was obtained and combined with ethyl acetate (3 mL). The solution was vortexed for 10 min and incubated at 40 °C. After 24 h, a portion of the ethyl acetate phase was mixed with 50 μL of a (1:1) mixture of (2M) hydroxyl amine and NaOH (3.5 M). Then, 90 μL of a (1:1) mixture of 10% ferric chloride in 4 M HCl was added to 95% ethanol. Then, the OD at 520 nm was measured, and the percentage of AHL inhibition was determined in the untreated culture [83].

#### 4.12.2. qRT-PCR

Total RNA extraction from the *P. aeruginosa* cultures was processed using the Direct-zol™ RNA Miniprep kit (Zymo Research Company, Tustin, CA, USA) following the manufacturer’s instructions. Isolated RNA was then used for complementary DNA synthesis using SensiFAST™ cDNA Synthesis Kit (Bioline Reagents, London, UK) according to the manufacturer’s steps.

The Bioline SensiFAST™ SYBR^®^ No-ROX Kit (Bioline Reagents, UK) workflow was used to quantify genes of interest in a technical triplicate manner for each sample, followed by a melting curve run and quality analysis. Genes of interest expression were normalized to the expression of 16S rRNA as a housekeeping gene using the 2^ΔCT^ formula. The primers (Forward and Reverse) used in qPCR are listed in Table 3.

### 4.13. Statistical Analysis

All experiments were performed in triplicate. Results were reported in the form of standard deviation, which was calculated using the Microsoft Excel 2009 software. The figures were prepared using GraphPad Prism (8.0). A one-way ANOVA was used to determine the significant difference between groups based on *P*-values as follows: * *p* < 0.05, ** *p* < 0.01, *** *p* < 0.001 compared to control untreated cells.

## 5. Conclusions

In conclusion, this study provides compelling evidence supporting the potential of the EO derived from *M. aurea* as a promising candidate for the treatment of infectious diseases associated with biofilm formation. The EO has demonstrated effective modulation of key virulence factors in *P. aeruginosa*, notably through disruption of the QS system. The observed efficacy could result from the presence of dominant active compounds or minor ingredients, possibly operating synergistically. This research not only underscores the therapeutic promise of the *M. aurea* derived EO but also suggests avenues for further exploration of its complex molecular mechanisms and potential applications in combating biofilm-associated infections.

## Figures and Tables

**Table 1:** List of compounds identified in *M. aurea* EO using GCMS.

Peak	Compound	Ki_cal_	Ki_let_	Conc (%)	Method of Identification
1	heptanol	970	935–977	0.6	RI, MS
2	β-pinene	978	959–982	0.3	RI, MS
3	1-octen-3-ol	980	964–986	0.3	RI, MS
4	α-terpinene	1010	1012–1026	0.4	RI, MS, Co-injection
5	limonene	1029	995–1039	0.2	RI, MS, Co-injection
6	3-octen-2-one	1037	1037–1048	0.5	RI, MS
7	phenylacetaldehyde	1043	1039–1049	0.5	RI, MS
8	(E)-2-octenal	1052	1049–1064	0.5	RI, MS
9	octanol	1074	1068–1078	0.3	RI, MS
10	terpinolene	1089	1081–1097	0.2	RI, MS
11	2-nonanone	1092	1090–1096	0.5	RI, MS
12	nonanal	1102	1087–1104	0.5	RI, MS
13	menthone	1148	1124–1155	0.3	RI, MS
14	hexyl butyrate	1190	1178–1194	0.5	RI, MS
15	caprylic acid	1191	1170–1192	0.2	RI, MS
16	octyl acetate	1200	1195–1215	1.0	RI, MS
17	(E)-2-decenal	1260	1234–1267	1.7	RI, MS
18	(E, E)-2,4-decadienal	1318	1312–1345	0.5	RI, MS
19	octyl isobutyrate	1345	1336–1348	0.3	RI, MS
20	α-copaene	1372	1364–1380	0.5	RI, MS
21	β-caryophyllene	1415	1394–1451	0.4	RI, MS, Co-injection
22	α-humulene	1455	1442–1488	0.7	RI, MS, Co-injection
23	trans-β-farnesene	1457	1456–1461	0.5	RI, MS
24	Germacrene D	1474	1468–1519	1.9	RI, MS
25	cuparene	1504	1498–1516	0.9	RI, MS
26	α-farnesene	1507	1505–1524	4.8	RI, MS
27	(E, E)-α-Farnesene	1507	1499–1511	1.5	RI, MS
28	β-bisabolene	1510	1489–1512	6.3	RI, MS
29	myristicin	1520	1509–1526	1.2	RI, MS, Co-injection
30	spathulenol	1576	1557–1580	0.5	RI, MS
31	humulene epoxide II	1606	1601–1620	0.7	RI, MS
32	isopropyl dodecanoate	1626	1614–1629	1.0	RI, MS
33	α-Bisabolol	1682	1680–1704	0.4	RI, MS
34	α-Bisabolol oxide A	1745	1672–1759	64.8	RI, MS
35	hexahydrofarnesyl acetone	1830	1827–1856	0.4	RI, MS
	Monoterpenes hydrocarbon			1.1	
	Oxygenated monoterpenes			0.3	
	Sesquiterpene hydrocarbons			17.5	
	Oxygenated sesquiterpenes			66.8	
	Others			10.1	
	Total			95.8	

**Table 2:** Minimum inhibitory concentration of *M. aurea* EO against *P. aeruginosa.*

Tested material	Inhibition zone (MIC)
*M. aurea* EO (1 mg)	12.8 ± 0.3 mm (1.25 mg/mL)
Erythromycin (10 µg)	10.33 ± 0.58
DMSO	0.0 ± 0.0

DMSO; dimethyl sulfoxide, MIC; minimum inhibitory concentration.

**Table 3:** Sequences of primers for 16S rRNA, *PhazA1*, *apraA*, *PelA,* and *Psl* genes.

Gene	Primer Sequence 5′-3′		References
16S rRNA	Forward	CAAAACTACTGAGCTAGAGTACG	[84]
	Reverse	TAAGATCTCAAGGATCCCAACGGCT	
*PelA*	Forward	CCTTCAGCCATCCGTTCTTCT	[85]
	Reverse	TCGCGTACGAAGTCGACCTT	
*PslA*	Forward	AAGATCAAGAAACGCGTGGAAT	[86]
	Reverse	TGTAGAGGTCGAACCACACCG	
*PhazA*	Forward	CGAACCACTTCTGGGTCGAGTGC	[87]
	Reverse	GGGAATACCGTCACGTTTTATTTGC	
*AprA*	Forward	GGCAATCCTGGTACCTGATCAA	[88]
	Reverse	AGCGTCTGGCGCCCGTAGTT	

## Data Availability

Data is contained within the article.
